# Quinquennium of Medicine and Surgery, 1906 to 1910

**Published:** 1911-12

**Authors:** 


**Quinquennium of Medicine and Surgery, 1906 to 1910.** Edited by J. W. BALLANTYNE, M.D. Pp. 411. Edinburgh: Wm. Green & Sons. 1911.

In vol. xxvii., p. 259, we gave a short notice of the last volume (No. X) of Green's *Encyclopedia and Dictionary of Medicine and Surgery.* It was then stated that the promises made in the Introduction had been accomplished. A medical dictionary had been incorporated with the *Encyclopaedia Medica,* and a supplementary volume was then being prepared, which is now before us. It has no preface, but it was intended to be a trustworthy guide to the really important additions which the past five years have given to our means of combating disease, deformity and death. It is arranged in dictionary form with many cross references. We can only pick out one or two topics for comment. Under the heading Tuberculosis we find that the work of the tuberculosis dispensary is being well recognised as the link between the Public Health Department and the various philanthropic agencies which are concerned in tuberculosis. Treatment by tuberculin receives a lengthy and complete notice, concluding as follows: "The fact that it has now overcome the prejudice which its use excited in the early nineties is perhaps the best proof that it is really a valuable remedy."

Under the heading Cerebro-spinal Meningitis we find a record of recent investigations of this comparatively new epidemic. All observers have confirmed the specificity of the diplococcus intracellularis meningitidis of Weichselbaum, which is easily found in the fluid withdrawn by lumbar puncture. Another noteworthy fact is that the spinal fluid always has a low opsonic and agglutinative power, in striking contrast with the high opsonic and agglutinative power which the blood serum develops. This " positive reaction " of the blood serum is so constant after the fifth day as to serve as a reliable diagnostic test. Much information is given as to the mode of infection, channel of entry (naso-pharynx), and spread of the disease, and the relation of epidemic to posterior basic meningitis is fully considered. The serum treatment appears to promise much, but whatever serum be used it must be injected into the spinal canal.

The article on Immunity, with a clear exposition of the theories of Ehrlich and Metchnikoff, is very complete. The account of Anaphylaxis shows the importance of its clinical recognition, whilst the diagnostic significance of the opsonic index and the various diagnostic serum tests both for tuberculosis and syphilis are well described.

Many useful illustrations and a full index conclude this well-printed work.

